# Epidemiology and clinical findings associated with enteroviral acute flaccid paralysis in Pakistan

**DOI:** 10.1186/1471-2334-7-6

**Published:** 2007-02-15

**Authors:** Mohsan Saeed, Sohail Z Zaidi, Asif Naeem, Muhammad Masroor, Salmaan Sharif, Shahzad Shaukat, Mehar Angez, Anis Khan

**Affiliations:** 1 Research Student, Department of Virology and Immunology, National Institute of Health, Islamabad, Pakistan; 2 Head of Department of Virology and Immunology; Principal Investigator, WHO Regional Reference Laboratory for Polio Eradication Initiative, National Institute of Health, Islamabad, Pakistan; 3 Virologist, WHO Regional Reference Laboratory for Polio Eradication Initiative, Department of Virology, National Institute of Health, Islamabad 45500, Pakistan; 4 Molecular Biologist, WHO Regional Reference Laboratory for Polio Eradication Initiative, National Institute of Health, Islamabad, Pakistan

## Abstract

**Background:**

Enteroviruses are among the most common viruses infecting humans worldwide and they are associated with diverse clinical syndromes. Acute flaccid paralysis (AFP) is a clinical manifestation of enteroviral neuropathy, transverse myelitis, Guillian-Barre Syndrome, Traumatic neuritis and many other nervous system disorders. The objective of this study was to understand the role of Non-Polio Enteroviruses (NPEV) towards this crippling disorder.

**Methods:**

Stool specimens of 1775 children, aged less than 15 years, suffering from acute flaccid paralysis were collected after informed consent within 14 days of onset of symptoms during January 2003 to September 2003. The specimens were inoculated on RD and L20B cells using conventional tube cell culture while micro-neutralization test was used to identify the non-polio enterovirus (NPEV) serotypes. Detailed clinical information and 60-days follow-up reports were analyzed for NPEV-associated AFP cases.

**Results:**

NPEV were isolated from 474 samples. The male to female ratio was 1.4:1. The isolation of NPEV decreased significantly with the increase in age. Cases associated with fever at the onset of NPEV-associated AFP were found to be 62%. The paralysis was found asymmetrical in 67% cases, the progression of paralysis to peak within 4 days was found in 72% cases and residual paralysis after 60 days of paralysis onset was observed in 39% cases associated with NPEV. A clinical diagnosis of Guillian-Barre syndrome was made in 32% cases. On Microneutralization assay, echo-6 (13%) and coxsackievirus B (13%) were the most commonly isolated serotypes of NPEV along with E-7, E-13, E-11, E-4 and E-30. The isolates (n = 181) found untypable by the antiserum pools were confirmed as NPEV by PCR using Pan-Enterovirus primers.

**Conclusion:**

The present study suggests that NPEV are a dominant cause of AFP and different serotypes of NPEV are randomly distributed in Pakistan. The untypable isolates need further characterization and analysis in order to determine their association with clinical presentation of a case.

## Background

Enteroviruses (genus Enterovirus, family Picornaviridae) are among the most common viruses infecting humans worldwide. Enteroviruses are associated with diverse clinical syndromes ranging from minor febrile illness to severe, potentially fatal conditions (e.g., aseptic meningitis, encephalitis, paralysis, myocarditis, and neonatal enteroviral sepsis) and could be linked with the development of some chronic diseases (e.g., type 1 diabetes and dilated cardiomyopathy) [1,2].

Serotypes of human enteroviruses have traditionally been classified into echoviruses, coxsackieviruses group A and B, and polioviruses. This traditional taxonomy was based on the associated disease in humans and animal model systems, sometimes resulting in overlaps between groups and difficulties with classification. As a result, beginning in the 1960s, newly discovered enteroviruses received a numeric designation (e.g., enterovirus 71) instead of being assigned to one of the traditional groups [1].

Current taxonomy takes into account molecular and biologic characteristics and divides human enteroviruses into four species (human enterovirus [HEV] A, B, C, and D) but keeps traditional names for individual serotypes [3]. Sixty-eight serotypes are included in the International Committee on Taxonomy of Viruses classification. The distribution of enteroviruses by species only partially corresponds to the groups in the traditional classification. Because molecular techniques of enterovirus typing are becoming increasingly available, new enteroviruses continue to be identified, and enteroviruses 79–101 have been recently described [4-12]. Echoviruses 22 and 23 have been reclassified as a new genus (Parechovirus) in Picornaviridae and are termed human parechoviruses 1 and 2, respectively. Although they belong to genetically and biologically distinct genera, human parechoviruses and human enteroviruses share many epidemiologic and clinical characteristics [3].

Various studies in India suggest the frequency of NPEV isolation from acute flaccid paralysis (AFP) cases from 20% to 54% [13-15]. Most of the AFP cases in United States of America were found to be caused by NPEV in the post vaccination era [16]. Several cases of paralysis were reported in association with enteroviruses, especially coxsackieviruses in Scotland [17]. Paralysis has also been reported in association with coxsackieviruses B2-B6, enterovirus 71 and echovirus types 3, 4, 6, 9, 11, 19 and 22 [18-20].

Enteroviruses grow well on HeLa cells, Hep-2 cells, human rhabdomyosarcoma cell line RD cells, MRC-5 cells, human embryonic kidney cells and buffalo green monkey kidney cells [21] but the RD cell line was found to be the most sensitive cell line for the isolation of enteroviruses [22]. Enteroviruses can be isolated from faeces, pharyngeal washings, cerebrospinal fluid (CSF), spinal cord, brain, heart, blood, conjunctivae and lesions of skin or mucous membrane. Retrospective evaluation of the relative utilities of different clinical specimens has proven that stool should be cultured for all patients suspected of NPEV infection [23].

Serotypes may be identified by reference antisera. The antiserum pools are issued in freeze-dried form to reference centres after formal request to WHO, Geneva [24]. The main objective to conduct this study was to identify the NPEV serotypes prevalent in Pakistan and their association with AFP cases.

## Methods

### a) Patients and specimens

The study was approved by Internal Review Board of National Institute of Health, Islamabad. All AFP cases in children aged <15 years were detected through active surveillance with a network of surveillance officers posted through out the country during January 2003 to September 2003. Two stool samples 24–48 hours apart from a total of 1775 AFP cases were collected within 14 days of onset of paralysis with the prior oral/verbal informed consent. Patient information was recorded on a standard questionnaire including demographic details, date of onset of paralysis and clinical presentation like fever at the onset of paralysis, symmetry of paralysis, location of paralysis and time duration for paralysis progression to peak. Specimens were transported to the Regional Reference Laboratory, National Institute of Health, Islamabad in good condition (cold chain maintained, container not leaking, good specimen quality and adequate quantity). Sixty-day follow-up reports were obtained through the same network.

### b)Virus isolation

The samples were treated with chloroform to remove bacteria and fungi and to dissociate virus aggregates. Conventional tube cell culture method was used for virus isolation in accordance with WHO recommendations [25,26]. Each specimen extract (0.2 ml) was inoculated in two cell culture tubes, one containing RD cells and other L20B cells, after aspirating the growth medium and replacing it with 1 ml maintenance medium. The tubes were incubated in the stationary sloped (5°) position at 36°C and examined daily for evidence of cytopathic effect (CPE). When complete CPE was obtained, the infected cells were harvested and kept frozen (-20°C) until typing.

### c) Microneutralization assay

The typing sera kit was provided by the National Institute of Public Health and the Environment, Bilthoven, the Netherlands (RIVM). The kit contained reference-typing sera against 21 of the 64 known human NPEV serotypes combined as nine antiserum pools. 50 μl of antisera was added to the appropriate wells of microtiter plate. Each isolate was tested in duplicate against all the NPEV antiserum pools using 10^-3 ^and 10^-4^dilutions. 50 μl of both the dilutions were dropped in respective wells and incubated at 37°C for one hour. After incubation, 100 μl of RD cell suspension was distributed into these wells and the plates were incubated at 37°C after covering with non-toxic sealer. Virus controls and cell controls were run along for comparison. The plates were examined daily, till the virus control showed 4^+ ^CPE. The identification was made by analyzing the pattern of inhibition of CPE by the antiserum pools.

### d) Polymerase chain reaction

In vitro amplification of the virus isolates was performed by PCR using pan-Enterovirus (Pan-EV) primers which were found untypable by neutralization test. The primer sequence was as follow

5'-ACACGGACACCCAAAGTAGTCGGTTCC-3'

5'-TCCGGCCCCTGAATGCGGCTAATCC-3'

The primers used for PCR amplification were selected from the highly conserved 5'non-coding region of the enterovirus and product was amplified by one step RT/PCR [27]. Non-infectious control RNA of pan-enteroviruses was used as positive control, culture supernatant from uninfected cells was used as negative control while buffer instead of samples was used as reagent control. Virus samples were diluted 1:4 in RNase free water. 1 μl diluted sample was added to 19 μl reaction mixture containing 250 μM of each dNTP, 2.5 ul of 10× PCR buffer and 40 pmol of each forward and reverse primers. The reaction mixture was first incubated at 95°C for 5 minutes and then chilled on ice for 5 minutes. Once tube contacts cooled, 5 μl mixture containing 2.0 mM MgCl_2_, 0.5 mM DTT, 0.2 units Placental RNase inhibitor, 0.07 units AMV reverse transcriptase and 1.5 units Taq polymerase was added. The tubes were placed in thermocycler (BIORAD I-cycler) and cycled as follows: 42°C for 20 min, 95°C for 3 min, 30 cycles at 95°C for 45 sec, 55°C for 45 sec 60°C for 45 sec, respectively. 114 bp sized band of the PCR amplified product was visualized under UV illumination on 10% Poly-acrylamide gel after ethidium bromide staining.

### e) Data analysis

Statistical analyses were performed using Epi Info version 6.0d [28].

## Results

### Epidemiology

**Figure 1:**
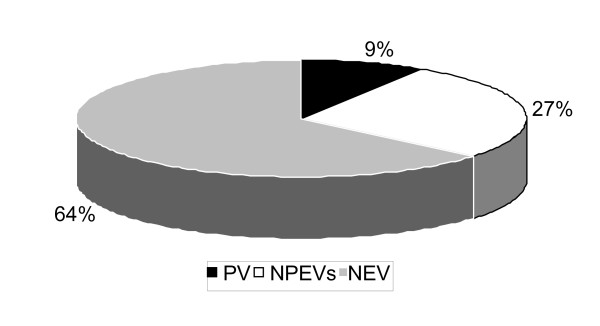
Enterovirus isolation rate from stool.

NPEV were isolated from 474 (26%) of a total of 1775 stool specimens collected during January 2003 to September 2003. The percentage of different virus isolates has been summarized (Fig. 1).

**Figure 2:**
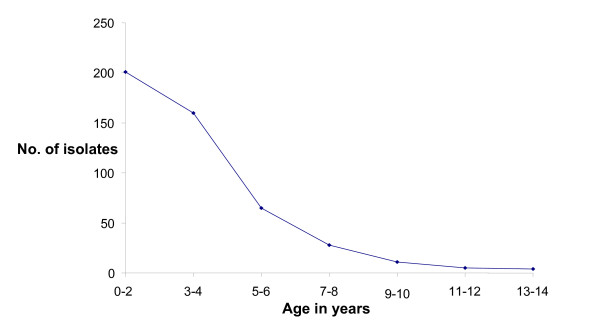
Age wise distribution of NPEV isolates.

A total of 398 (84%) NPEV isolates were reported from children younger than 5 years. The detection of NPEV in AFP cases decreased with the increase in age (Fig. 2) and statistical analysis showed strong negative correlation (r = -0.913) between age and detection of NPEV. The analysis of the results by chi-square test showed high significance (p = 0.002). Out of total 474 isolates, 276 (58%) were isolated from males, with a male to female ratio of 1.4: 1. The variation was not found significant (χ^2 ^= 0.545 and p = 0.4602). Association between age and sex was checked by applying chi square test and it was found that no association existed between these two attributes (χ^2 ^= 0.098 and p = 0.99).

### Virology

All the NPEV isolates were typed by micro-neutralization assay. There were 220 (46%) Echoviruses, 63 (13%) Coxsackievirus B, 10 (2%) Coxsackievirus A9 and 181 (38%) untypable viruses (Table 1). Echo-6 and Coxsackievirus B were the most commonly isolated serotypes. The analysis of the geographical distribution of the different serotypes showed that NPEV serotypes are randomly distributed in almost all parts of the country. All of 181 isolates found untypable by microneutralization assay were amplified by RT-PCR using pan-EV primers and all of these isolates were confirmed as enteroviruses.

**Table 1:** Identification of different serotypes identified by Microneutralization test

**Serotype**	**No. of Isolates**	**Percentage of Total**
ECHO 6	63	13
Cox B	63	13
ECHO 7	42	9
ECHO 13	35	7
ECHO 11	35	7
ECHO 4	14	3
Cox A9	10	2
ECHO 1	7	1
ECHO 25	7	1
ECHO 30	6	1
ECHO 20	6	1
ECHO 2	5	1
Untypable	181	38

Total	474	100

### Clinical data

The case investigation forms of 190 NPEV-associated AFP cases were analyzed for clinical characteristics. 116 (61%) out of 190 patients were found to have fever at the onset of paralysis. No correlation was found between age and presence of fever (r = 0.02). The association between sex and presence or absence of fever was statistically found insignificant (χ^2 ^= 0.4183, p = 0.5177). The paralysis was found asymmetrical in 128 patients (67%). Legs were affected in 95% patients while arms in 40%. The facial palsy was observed only in 19 patients (10%). In 137 patients (72%) the paralysis progressed to peak within 4 days of onset, the progression took more than 4 days in 16 patients (8%) while no credible information could be obtained for 37 patients (20%). The most common preliminary diagnosis for AFP cases with NPEV isolates was Guillain-Barre' syndrome (33%) followed by viral neuropathy (Table 2). On 60 day follow up after the onset of paralysis, residual paralysis was still present in 74 patients (39%) while 23 patients (12%) died within 60 days following paralysis.

**Table 2:** Clinical diagnosis of cases reported with paralysis

**Clinical diagnosis**	**No. of Cases**	**Percentage of total**
Guillain-barre Syndrome	62	33
Viral Neuropathy	51	26
Infantile Hemiplegia	30	14
Traumatic Neuritis	17	8
Meningitis	9	4
Encephalitis	3	1
Arthritis	3	1
Transverse Myelitis	2	1
Unknown	23	12

Total	190	100

## Discussion

The work presented here is the first report on prevalent serotypes of NPEV in Pakistan along with their epidemiology and clinical characteristics. The work also reflects that isolation of NPEV from AFP cases is common in Pakistan. The findings are in accordance with the studies conducted in India, where NPEV were isolated from 34% of AFP cases. The frequency of NPEV was higher because only the children less than 4 year of age were recruited. Another study in India had isolated NPEV from 20% of acute poliomyelitis cases [13,14].

Enteroviral infections are more prevalent in children than in adults [29-31]. The age distribution of enterovirus-associated illness in a 10-year surveillance report from the United States recorded that most of the cases were reported in young children [32]. The present study shows that the age of children with NPEV-associated AFP ranged from 1 to 14 years and that the isolation of NPEV decreased significantly with the increase in age. Similar pattern was observed by Morens who analyzed the reports to the CDC on isolation of NPEV for the years 1971–1975 and found that the incidence of reported isolations decreased with increasing age [31].

Serotyping of the NPEV isolated from the patients of AFP in India showed that Echovirus 6, 11, 9 and coxsackievirus B were the most frequent serotypes of NPEV [14]. In the present study, 38% isolates could not be identified by the antiserum pools provided by WHO. The pools contained antibodies against only 21 of the 68 known human enterovirus serotypes, so their failure to neutralize a given isolate could have been due to the absence of homologous antibodies in the pools used, virus aggregation or presence of a virus mixture [15]. Many enterovirus isolates could not be typed by the microneutralization test during a study of epidemiology of different enterovirus serotypes in Netherlands [33].

With worldwide Poliovirus surveillance and the recent development of new molecular tools for enterovirus detection and identification, more interest is being given to NPEV especially in polio free countries. As the endemic countries harbouring circulation of wild polioviruses also happen to be developing countries therefore the utility of resources remains low on research oriented activities. A major chunk of resources is still being utilized for eradication activity in countries like Pakistan. Standard methods for enterovirus detection and identification are based on virus isolation on cell culture followed by serotyping the isolated viruses by microneutralization assays using pools of serotype specific antisera [34]. This procedure is time consuming and labour intensive and the availability of specific antisera gradually becomes restricted. Several techniques for rapid detection of the enterovirus genome in clinical samples, most of them based on PCR amplification in the 5' non-coding (5' NC) of the genome have been developed. In this study similar method was used to identify the isolates (n = 181) that could not be serotyped by enterovirus-specific pools available in the laboratory. However such methods do not allow serotype identification and genetic characterization of the detected viruses beyond the genus level [35]. Thus when information on the serotype is needed, virus isolation on cell culture remains the most appropriate technique. To overcome the problems related to the specific antisera, attempts have been made to develop new methods for typing enteroviruses by PCR amplification and partial sequencing in the VP1 region of the genome [9-11].

Fever at the time of onset of paralysis is one of the cardinal signs of poliomyelitis but cases of AFP with NPEV isolates were also reported to have fever at the time of onset. Fever was a major symptom in 53% AFP cases associated with NPEV in India [14]. The presence of fever in almost two third cases in the present study may be due to the fact that most of the time case was reported from hospital where the paralysis had reached its peak. The clinical significance of fever at the onset of paralysis along with rapid progression of asymmetrical paralysis helps in the determination of neuropathies caused by NPEV. 12% patients died within 60 days of the onset of paralysis owing to the severity of disease. No association could be found out between death of patient with age at infection or NPEV responsible.

'The progression of paralysis to peak within 4 days of onset' was recorded in 52% of NPEV-associated AFP cases in Americas with residual paralysis in 24% cases [16]. In the present study, 'the progression of paralysis within 4 days of onset' demonstrated higher percentage reflecting the high severity of disease but its association with low immunity status or lack of proper treatment could not be determined unless further studies are conducted.

## Conclusion

The present study suggests that after the eradication of poliomyelitis, AFP cases negative for wild poliovirus but positive for NPEV will continue to be detected. The data also confirms that NPEV circulation is common and isolates may be obtained from persons with AFP whose clinical findings do not resemble poliomyelitis. The results of this study indicate that characterization of NPEV isolates could provide better understanding of the epidemiology of NPEV causing paralysis. Data generated from this study will help future studies on NPEV serotypes circulating in Pakistan and to formulate more effective strategies in view of post eradication era. A better knowledge of the transmission and the implications of NPEV in diseases may also justify the future studies on their molecular epidemiology.

## Competing interests

The author(s) declare that they have no competing interests.

## Authors' contributions

MS carried out all the experimental work. AK assisted during the cell culture work. SS, SS and MA assisted with the experimental work particularly PCR. MM helped in data interpretation. SZZ helped design the study and supervised the work. AN wrote the manuscript. All the authors have read and approved the final manuscript.

## Pre-publication history

The pre-publication history for this paper can be accessed here:


